# Whole Genome Sequencing for Determining the Source of *Mycobacterium bovis* Infections in Livestock Herds and Wildlife in New Zealand

**DOI:** 10.3389/fvets.2018.00272

**Published:** 2018-10-30

**Authors:** Marian Price-Carter, Rudiger Brauning, Geoffrey W. de Lisle, Paul Livingstone, Mark Neill, Jane Sinclair, Brent Paterson, Gillian Atkinson, Garry Knowles, Kevin Crews, Joseph Crispell, Rowland Kao, Suelee Robbe-Austerman, Tod Stuber, Julian Parkhill, James Wood, Simon Harris, Desmond M. Collins

**Affiliations:** ^1^AgResearch, Hopkirk Research Institute, Palmerston North, New Zealand; ^2^AgResearch, Invermay Agricultural Centre, Mosgiel, New Zealand; ^3^TBfree NZ, Wellington, New Zealand; ^4^TBfree NZ, Christchurch, New Zealand; ^5^TBfree NZ, Hamilton, New Zealand; ^6^TBfree NZ, Dunedin, New Zealand; ^7^TBfree NZ, Palmerston North, New Zealand; ^8^Aquaculture Veterinary Services Ltd., Clyde, New Zealand; ^9^University College Dublin School of Veterinary Medicine, Dublin, Ireland; ^10^Royal (Dick) School of Veterinary Studies and Roslin Institute, University of Edinburgh, Edinburgh, United Kingdom; ^11^Diagnostic Bacteriology Laboratory, National Veterinary Services Laboratories, U.S. Department of Agriculture, Animal and Plant Health Inspection Service, Veterinary Service, Ames, IA, United States; ^12^Wellcome Sanger Institute, Wellcome Genome Campus, Cambridge, United Kingdom; ^13^Department of Veterinary Medicine, University of Cambridge, Cambridge, United Kingdom

**Keywords:** *Mycobacterium bovis*, molecular fingerprint, whole genome sequencing, New Zealand, bovine tuberculosis control, epidemiology

## Abstract

The ability to DNA fingerprint *Mycobacterium bovis* isolates helped to define the role of wildlife in the persistence of bovine tuberculosis in New Zealand. DNA fingerprinting results currently help to guide wildlife control measures and also aid in tracing the source of infections that result from movement of livestock. During the last 5 years we have developed the ability to distinguish New Zealand (NZ) *M. bovis* isolates by comparing the sequences of whole genome sequenced (WGS) *M. bovis* samples. WGS provides much higher resolution than our other established typing methods and greatly improves the definition of the regional localization of NZ *M. bovis* types. Three outbreak investigations are described and results demonstrate how WGS analysis has led to the confirmation of epidemiological sourcing of infection, to better definition of new sources of infection by ruling out other possible sources, and has revealed probable wildlife infection in an area considered to be free of infected wildlife. The routine use of WGS analyses for sourcing new *M. bovis* infections will be an important component of the strategy employed to eradicate bovine TB from NZ livestock and wildlife.

## Introduction

Efforts to control bovine tuberculosis (TB) in domestic livestock in New Zealand (NZ) are driven by the zoonotic risk of the causative agent *Mycobacterium bovis* and its possible impacts on international trade (1, 2). Although in many countries bovine TB has been controlled successfully with test and slaughter strategies and movement restriction, control is particularly challenging in countries like NZ in which there is a wildlife reservoir of infection (1, 3). In Britain and Ireland the Eurasian badger harbors and spreads *M. bovis*, in France the wild boar, in Michigan and Minnesota in the USA, deer, and in NZ the brush-tail possum, [reviewed in (4)]. Effective control under these circumstances involves not only test and slaughter and movement control but also knowledge and control of the infection status in wildlife (3, 5). The challenges imposed in different parts of the world by these varied sources have been reviewed (6). Despite the challenging circumstances imposed by its wildlife reservoir, the control of bovine TB in NZ has recently been re-evaluated and there are now ambitious goals of achieving TB free livestock and wildlife by 2026 and 2040, respectively (2, 7).

Molecular methods provide a means to detect and characterize the spread of pathogens in both domestic livestock and in wildlife populations (3–5, 8). Studies in NZ that employed an early DNA fingerprinting assay that compared the restriction pattern of DNA digests (Restriction Endonuclease Analysis REA typing) of *M. bovis* isolates, demonstrated that livestock and wildlife in the same regions tended to share the same types and thus helped to define the role of wildlife in the spread of bovine TB in New Zealand (1, 9, 10). REA typing was used routinely for over 20 years to efficiently guide wildlife control measures and to aid in tracing the sources of infections that resulted from movement of livestock (10). In other parts of the world, comparison of the direct repeat region of the *M. bovis* chromosome by a process called spoligotyping, and a more sensitive PCR based method that compares repeated sequences at different sites in *M. bovis* genomes, [Variable Number Tandem Repeat (VNTR)] have been used for monitoring the genotypes of isolates from wildlife and livestock, providing insight into the types and spread of *M. bovis* (10–14). Because VNTR was simpler to perform and interpret than REA and was almost as discriminating, the REA method was replaced in NZ by VNTR in 2012 (15). VNTR fingerprint typing is routinely employed in NZ to determine the source of new livestock infections and the types carried by wildlife. In many cases VNTR clearly identifies the regional source of new infections, but it is of less use in cases where the same type is widespread in one or more regions of the country.

Recent advances in DNA sequencing have facilitated the routine comparisons of entire bacterial genomes [whole genome sequencing (WGS)] for determining the source of bacterial infections and this technology shows promise in aiding bovine TB control including situations that are complicated by wildlife reservoirs (4, 16–22). The single nucleotide polymorphism (SNP) lineages that result from WGS are far superior to the “types” that come from comparing a small number of sites in Spoligo or VNTR typing analyses. There are typically tens to hundreds of SNPs common to a major branch, and 10 s of SNPs common to sub-clusters in each branch. Because so many more similarities and differences are considered in comparisons of lineages, there is less chance for misinterpretation of the relationship between isolates than when typing by VNTR (18, 23). In addition, these lineages provide information about shared common ancestors that is not always obvious by VNTR and spoligotyping. When coupled with knowledge of how quickly these bacteria accumulate new SNPs, this information can provide temporal clues about the arrival and divergence of types, which can greatly aid epidemiological investigations.

The rigor of WGS for elucidating phylogenetic relationships in NZ cycles was demonstrated with an analysis (24) performed on 296 NZ genomes that were available at the time. Four clades were identified and shown to have significant clustering by both REA type and by region but to lack significant clustering by host. These results verified the regional localization of types and rapid switching between wildlife and livestock hosts that was suggested by REA typing. With the extra resolution provided by WGS there were numerous instances where isolates that had identical REA types could now be distinguished. Analysis by a Bayesian approach (Bayesian Evolutionary Analysis by Sampling Trees BEAST) (25) on a subgroup for which there were an adequate number of wildlife and livestock isolates from one clade, that were spread over time, indicated that although there was significant variation, *M. bovis* in infected animals in NZ was accumulating mutations in a clocklike manner at a rate of 0.53 (2.5% Lower: 0.22, 97.5% Upper: 0.94) events per genome per year. The most recent common ancestor (MRCA) to this group was estimated to have been circulating in 1859 (2.5% Lower 1525 97.5% Upper 1936) which agreed with the time when *M. bovis* was likely to have been introduced into NZ in cattle imported directly and indirectly (via Australia) from the UK (26). This study provided convincing evidence that the enhanced resolution from WGS had potential to aid in more precisely determining whether new infections were from persistence or the introduction of infection into NZ livestock and wildlife populations.

Through partnership and contracted work with TBfree and collaborations with Wellcome Sanger Institute, the Wellcome Trust University of Glasgow, United States Department of Agriculture (USDA), Animal Health and Veterinary Laboratories Agency (AHVLA), Landcare Research and Massey University, we have developed a database with over 700 WGS entries of important NZ *M. bovis* types, and a data processing method that identifies robust SNPs that differ from a reference genome and compares these SNPs to those detected in other isolates. This information has helped to precisely define the lineage of NZ *M. bovis* types and has facilitated accurate determination of the source of new infections. Our WGS database has been enriched in recent years by characterizing additional isolates from recent herd breakdowns and outbreaks and the characterization by the WGS of REA and VNTR types that were once prevalent in NZ. Here we demonstrate the suitability of WGS for routine surveillance with three investigations into NZ. *M. bovis* outbreaks in which genetic relatedness of the isolates were determined by comparing these novel SNP lineages to others in the database. The benefits of WGS over REA and VNTR typing methods in each case are discussed.

## Materials and methods

The AgResearch *M. bovis* archive has over 8000 NZ isolates that were cultured between 1985 and 2018, from livestock and wildlife suspected of *M. bovis* infection during the post-mortem examination performed as part of routine surveillance. Conventional microbiological tests [described in (27)] were used to positively identify *M. bovis* infection. The WGS database has been assembled by characterizing isolates from the archive selected to provide a representative sample of the *M. bovis* population circulating in cattle and wildlife across NZ between 1985 and 2018. Most isolates that were characterized by WGS were previously either REA typed (10) or VNTR typed (15) and in some cases were typed by both methods. Culture and DNA isolation was performed either at the AgResearch Wallaceville or the AgResearch Hopkirk sites in level 3 containment facilities, adhering to the biosafety guidelines for these procedures outlined in the AgResearch containment facility manual. A total of 783 isolates; 417 bovine, 112 ferret, 106 possum, 72 pig, 67 cervine, 3 feline, 2 stoat, 1 hedgehog, and 1 human isolate were characterized by WGS. Selected isolates were cultured in Tween albumin (TAB) media from frozen stock and DNA was prepared by CTAB extraction essentially as described in (28). DNA submitted for sequencing at the New Zealand Genomic Limited facility at Massey University in NZ (NZGL) and the United States Department of Agriculture (USDA) was additionally purified by digestion with 20 mg/ml RNAse after lysozyme treatment, and with a phenol chloroform isoamyl alcohol extraction after incubation in N-cetyl-N,N,N-trimethyl ammonium bromide (CTAB). DNA library preparation and genome sequencing were performed either at the USDA facility in Ames Iowa USA, at the NZGL facility at Massey University in NZ, or at The Wellcome Trust Glasgow facility in the UK, on an IIlumina MiSeq instrument, with 2 X 250 bp paired-end reads or at the Wellcome Sanger Institute (Cambridge, UK) on an Illumina HiSeq instrument with 2 × 150 bp paired-end reads.

Raw genomic data were trimmed using the DynamicTrim algorithm (v2.0, default settings) in SolexaQA software (29) and mapped to the original UK reference genome (NC_002945.3, AF2122/97) (30) using the Burrows-Wheeler Aligner (BWA)-MEM algorithm (v0.7.9a-r786 with -M setting) in the BWA alignment tool (31). From the resulting alignments, reads that mapped to more than one location in the genome (SAM flags > = 256) were removed. Results were further processed with SAM tools software (32) (v0.1.19-44428cd with settings view -q 30; then rmdup -S) to remove low quality mappings and PCR duplicates. Indels and non A/C/T/G reference alleles were ignored, bcftools (v0.1.19-44428cd, with setting view -N -I -cvg). Subsequently a minimum alignment quality of 80, a minimum total depth per SNP of 10, a maximum reference allele count per SNP of 2, a maximum FQ value of −55, and a reference to alternative allele ratio of at least 0.9 was enforced. Multiinter from the bedtools suite (v2.17.0) was used to generate a final list of potential genomic differences. SNPs detected in regions that are not well characterized by this methodology, (33) such as PE PGRS regions, IS elements, and poorly covered regions were excluded from the analyses via VCF software (34) (v0.1.12b). Poorly covered regions were defined by comparing 344 genomes with an average coverage of 45X or higher. Regions from which SNPs were excluded and also individual SNPs that were excluded from all of the genomes because they were poorly covered in some of the genomes are listed in Supplementary File 1. SNPs that were determined to be of high quality when detected in genomes with 45X or higher coverage and detected but filtered from more poorly covered genomes were added back to the filtered VCF files of the poorly covered genomes. The remaining core SNPs were processed together to produce concatenated alignments, which were compared in order to define the phylogenetic relationship of the isolates. A *Mycobacterium caprae* genome (strain 09-0454) is included as an out-group to root these comparisons. Average coverage and *in silico* spoligotyping were determined with vSNP software / https://github.com/USDA-VS/vSNP using the recently amended UK reference NC_002945.4 (35).

The relationship between isolates that are shown here were determined by BioNJ phylogenetic trees with 100 replicates, using a Jukes and Cantor model with SeaView 4 software (36) and also with RAxML software (37) (version raxmlHPC-PTHREADS-SSE3) with 1000 replicates using a GTRGAMMAI model. BioNJ and RAxML Phylogenetic trees were compared side by side using Phylo.io software (38) and were displayed and colored for other Figures using FigTree v1.4.2 software (39). Distance matrices were generated from concatenated SNP sequences with the Muscle Aligner (40) in the Geneious software package and were colored using the color scale formatter in Excel or alternatively with an R script that uses R's Gplot package to create heat maps via the heatmap.2() function. Global distributions of the four major NZ spoligotypes were obtained with the similarity search tool at the Pasteur-Guadeloupe website http://www.pasteur-guadeloupe.fr:8081/SpolSimilaritySearch/ (41).

## Results

**Figure 1:**
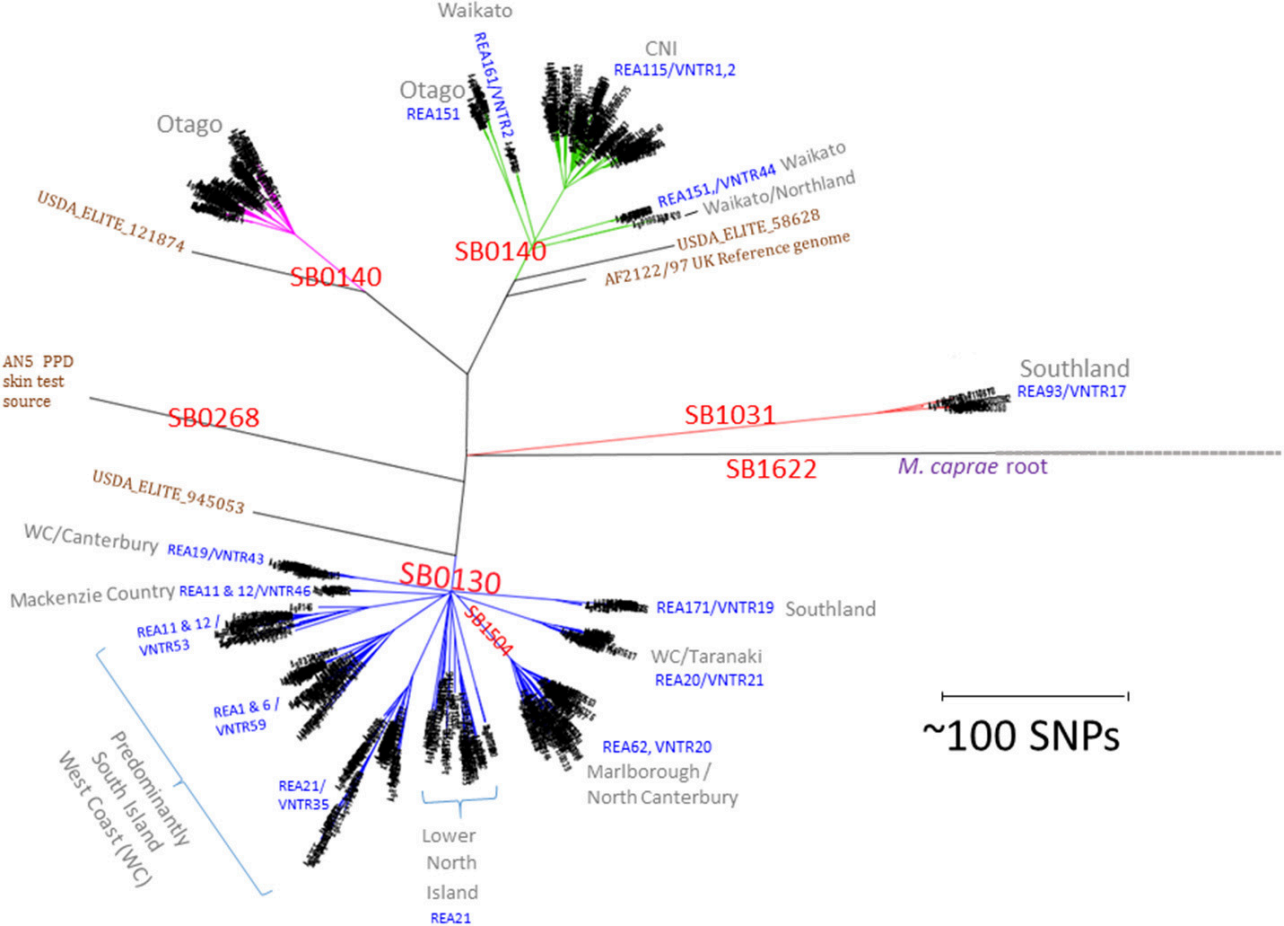
NZ *M. bovis* types. Radial Maximum Likelihood (ML) Phylogram illustrating the relationship of NZ *M. bovis* isolates in the NZ WGS database. The scale bar indicates the approximate distance in SNPs between isolates. “SB” numbers labeled in red are internationally recognized spoligotypes based on differences in the DR/CRISPR region. The REA and VNTR types listed in blue text are the predominant REA type(s) and or VNTR types in the indicated cluster and they are predominant in regions listed in gray. Overseas isolates that are included for comparison are labeled in brown: the UK reference (AF2122/97), the UK strain commonly used as a source of PPD (AN5) and 3 USDA ELITE strains (58628, 945053, and 121874). The branches in the four NZ clusters are colored differently to highlight the distinction from other branches.

A total of 782 *M. bovis* genomes were used here as a basis for comparison of NZ breakdowns and outbreaks. The alignment length for this selection of isolates was 8261 sites. Metadata, coverage statistics and the *in silico* spoligotyping results for these isolates are listed in the spreadsheet in Supplementary File 2. The time span for these isolates is 30 years from 1988 to 2018 and includes representatives of important types from throughout the North and South Island. The relationship of prevalent NZ *M. bovis* types is illustrated by the SNP phylogeny that was generated by maximum likelihood analysis in Figure 1 and is compared to a phylogeny determined by the BioNJ distance method in Supplementary File 3. Several genomes from overseas *M. bovis* isolates, including the PPD strain AN5, and three isolates from the USDA elite collection (05_8628, 94_5053, and 12_1874) are included to aid with these comparisons. The 4 distinct branches that were initially detected (24) were also evident in this larger group, and each clade was shown here to share more recent common ancestors with overseas isolates than with other NZ isolates. The same relationship was evident by a BioNJ distance analysis, perhaps reflecting the clonal, primarily non-recombining nature of NZ *M. bovis* evolution (see Supplementary File 3a). As was seen in the initial characterization (24) of a subset of these isolates, WGS results for this larger group of isolates corroborate findings from previous REA and VNTR typing studies which revealed that distinct types predominate in different parts of NZ (9, 10, 15).

**Figure 2:**
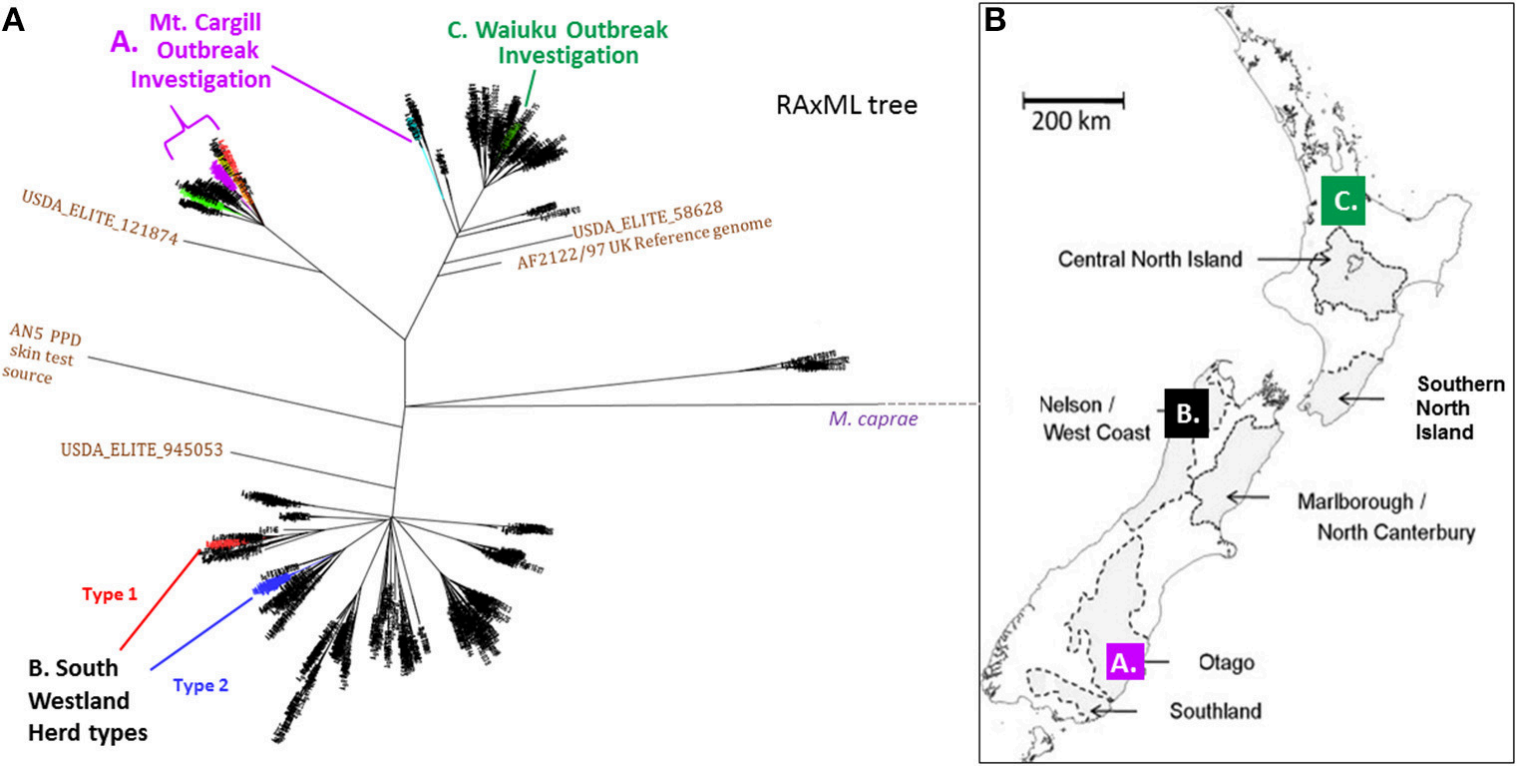
**(A)** NZ *M. bovis* Radial ML Phylogram illustrating the Phylogenetic relationship of the isolates that will be discussed in more detail below. (A) Mt. Cargill investigation isolates-These isolates cluster in the Otago clade and are colored the same as in the boxed sub-groups in Phylogram in Figure 3a. (B) South Westland breakdown types- The two types isolated from the herd in South Westland and the closest relatives (shown in SNP tables in Figure 4B) are colored differently; the breakdown type 1 isolate clusters in the VNTR53 group and it and its closest known relatives are colored red, the breakdown type 2 isolates clusters in the VNTR59 subgroup and it and its closest known relatives are colored blue. (C) Waiuku outbreak- isolates cluster in the CNI clade and outbreak isolates described in the SNP table in Figure 6B are colored green. **(B)** Map of NZ indicating vector risk areas (shaded regions) and illustrating geographical source of the isolates from the three outbreak investigations described below. Colored squares on the map indicate the regions of the discussed outbreak investigations. The three different investigations that will be discussed below are indicated by letters and color in the Phylogram in **(A)** and the map in **(B)**.

*In silico* spoligotyping revealed that most of the NZ isolates in the database have spoligotypes that were common in the UK when cattle were imported into NZ in late 1860s (42), 34% were SB0130 and 41% were SB0140. Two other prevalent spoligotypes that were characterized extensively were SB1504, (121 isolates 15%) a type that is endemic in the Marlborough North Canterbury region of the South Island and SB1031 (23 isolates 3%) a type that is endemic in Southland in the South Island (see the map presented in Figure 2B) and was once prevalent in Australia (43). The Global distribution of these types is illustrated in the Supplementary File 4. Although SB0130, SB0140 and SB1031 have been isolated in other parts of the world (43), SB1504 has so far only been detected in NZ, suggesting either that it evolved from a different type just prior to becoming established in NZ or that other global sources of this type have not yet been discovered.

The phylogenetic relationship and geographical source of the three outbreaks investigations that will be discussed below are described in the phylogeny in Figure 2A and map in Figure 2B. Infections that were investigated were from (A) Mt. Cargill in Otago, (B) South Westland in the South Island, and (C) Waiuku in the Central North Island (CNI).

### Mt. Cargill outbreak

An investigation of isolates from the Mt. Cargill region of the South Island was carried out to aid in determining the source of this recent infection, which appeared to have spread throughout the Mt. Cargill region within 1–2 years. We analyzed three groups of isolates: (i) isolates from recently infected cattle (9 isolates), farmed deer (2 isolates) and wildlife (14 isolates) in the region; (ii) a selection of isolates (5 cattle, 5 wildlife) from the AgResearch strain archive that had come from sources within 15 km of the outbreak; and (iii) a group of recent cattle isolates with similar types (AgR1665 type VNTR104, AgR1689 type VNTR135 and AgR1669 VNTR27) to those found in the Mt. Cargill region that had come from outside Mt. Cargill. All isolates were characterized by WGS in order to determine if this outbreak was from local wildlife reinfection, or from introduction of a different type into the region. Also shown in the accompanying figures are the relationship of these outbreak investigation isolates to previously characterized Otago isolates (AgR96, AgR707, AgR703, AgR726, AgR734, AgR51, AgR76, and AgR53) in the WGS database.

**Figure 3:**
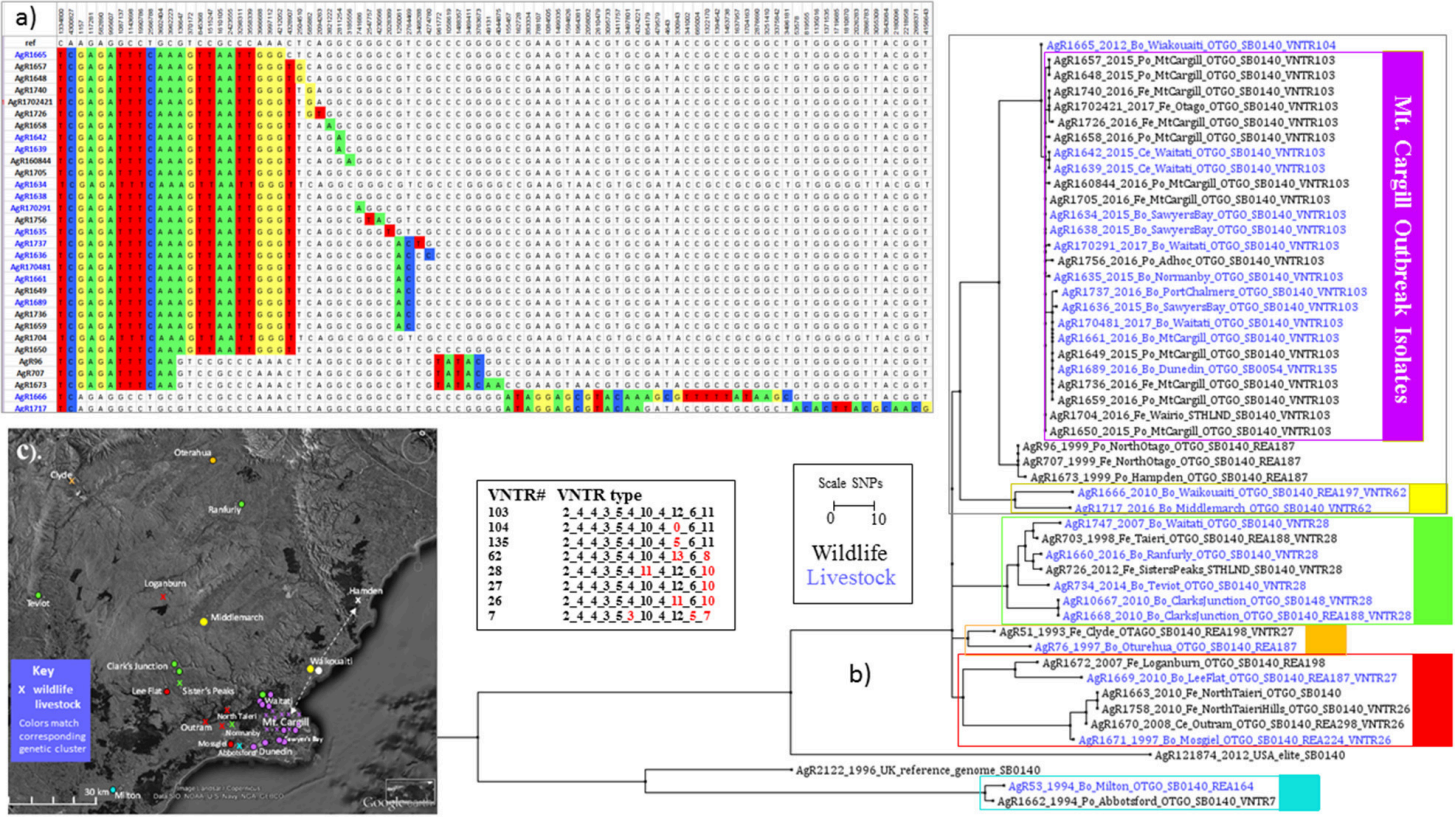
Mt. Cargill Outbreak Investigation. Genetic and spatial relationship of *M. bovis* isolates from southeast Otago. **(a)** SNP table and Phylogram for selected isolates from the Otago cluster. The SNP table illustrates SNP differences in outbreak isolates and their closest known relatives. Chromosomal positions in genomic reference NC_002945.3 are listed across the top. The DNA base found at the indicated chromosomal position in the reference is listed in the next line. DNA bases in the table are colored to indicate differences from the reference genome. **(b)** Square ML Phylogram. The scale bar indicates the distance in SNPs between isolates displayed in the phylograph. Livestock metadata in the tree (_Bo_ for bovine _Ce_ for cervine) is colored blue and wildlife metadata (_Po_ for possum, _Fe_ for ferret), is colored black. The numbers for the listed VNTR types are the number of repeats at the 11 loci as described in Price-Carter et al. (15): Miru40_EtrD_EtrC_EtrE_NZ2_QUB18_QUB11a_QUB26_DR2_DR1_QUB3232. Red numbers in this VNTR table indicate differences from the outbreak type VNTR103. **(c)** Map of sources of isolates shown in the phylogenetic tree in **(b)**. Symbols on the map indicate the approximate regional sources and are colored to match the genetic cluster of the isolate as indicated by the boxes in **(b)**. The arrows on the map in **(c)** indicate the proposed direction of the spread of this infection based on WGS results.

Results of this investigation are shown in (a) SNP Table and (b) the Phylogram (C) the Map in Figure 3. When WGS data for Mt Cargill outbreak isolates was compared to WGS data for the other isolates that were characterized for this investigation, the Mt. Cargill outbreak isolates (boxed in purple in Figure 3a) shared their most recent common ancestors with livestock and wildlife isolates from more than 20 km north of Mt Cargill and were more distantly related to the other examined types that were prevalent in the nearest known wildlife/domestic stock cases from west of Mount Cargill. The closest known relative to the outbreak was a 2012 isolate (AgR1665) from Waikouaiti (see the SNP table in Figure 3a). AgR1665 was missing one SNP that was common to the outbreak isolates, a C to T change at position 4328907 in the reference genome, but had the 11 others that were common to the outbreak cluster. Using the mutation rate estimate from Crispell et al. [0.53 (2.5% Lower: 0.22, 97.5% Upper: 0.94)] this suggests that this likely precursor and the Mt. Cargill outbreak lineage diverged from a common ancestor approximately 2 (3–7) years prior to when the 2012 precursor isolate was detected. The next closest known relatives were several wildlife isolates from northern Otago (AgR707, AgR96, and AgR1673). Mt Cargill isolates shared 10 common SNPs with these wildlife isolates. The Mt. Cargill and North Otago wildlife isolates shared 2 SNPs with recently characterized isolates from Northern Otago (AgR1717 and AgR1666, boxed in yellow in the phylogenetic tree in Figure 3b and indicated by yellow symbols on the map in Figure 3c). Both groups had acquired numerous SNPs (>20) since diverging from their common ancestor. These results provide evidence to suggest that this type had prevailed in Northern Otago for many years. Supplementary File 3b compares the relationship of these isolates by maximum likelihood and BioNJ distance analyses, and the conclusions drawn about the relationship of these isolates was the same regardless of the phylogenetic method used for the analysis perhaps because of the many SNPs that were common to the outbreak isolates and their closest known relatives. Although these results did not rule out the possibility that the infection was circulating undetected in the Mt Cargill region previous to the outbreak, our WGS comparison of outbreak isolates to common wildlife and livestock types suggest that this infection is more likely to have moved into Mt. Cargill from wildlife or livestock from the north than to have come from local wildlife. A direction of the spread of this infection based on these data is indicated by the arrows on the map in Figure 3c.

Although in most cases VNTR types of these isolates correlated well with SNP sub-clusters, since the closest relative, a 2012 Waikouaiti cattle isolate (AgR1665) had a slightly variant VNTR type (see the VNTR104 types tabulated in Figure 3b), if the Mt. Cargill outbreak investigation was based solely on VNTR results the relevance of this isolate to the outbreak would be much less evident than it is from the SNP lineage. This tree also illustrates how the SNP lineage determined by WGS defines the relationship between early isolates that were originally characterized only by REA to later isolates that were originally characterized only by VNTR, which can be very helpful when trying to understand the source of new infections.

### South Westland TB infected herd

**Figure 4:**
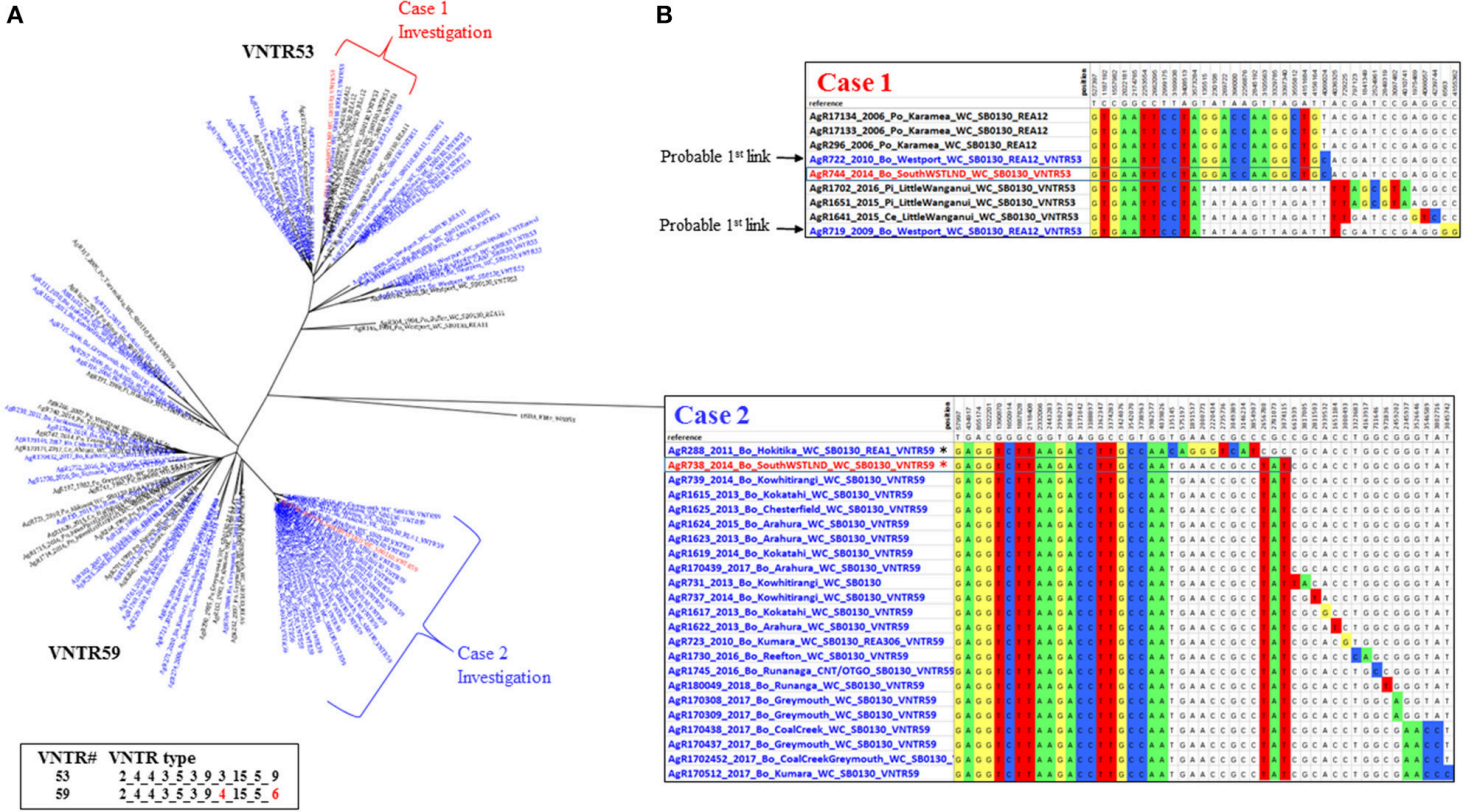
Multiple South Westland herd infections. **(A)** Radial ML Phylogram illustrating the genetic relationship of the two types of *M. bovis* isolates detected during a South Westland breakdown investigation, to other type VNTR59/REA1/REA6 and VNTR53/REA11/REA12 *M. bovis* isolates in the database. Metadata for isolates from this herd are colored red, other livestock metadata are colored blue and wildlife metadata black. Brackets indicate close relatives of the breakdown isolates and are also described in the SNP table in **(B)**. Also shown are the two different VNTR types, with numbering as described in the legend for Figure 3. **(B)** SNP tables illustrating the relationship of each type to its closest relatives. The coloring and numbering in this tables is as described in Figure 3. SNPs detected in the case 1 and in case 2 isolates are boxed within the table. The asterisk in the case 2 table indicates an isolate (AgR288) that was ruled out as a possible source of infection by this investigation.

**Figure 5:**
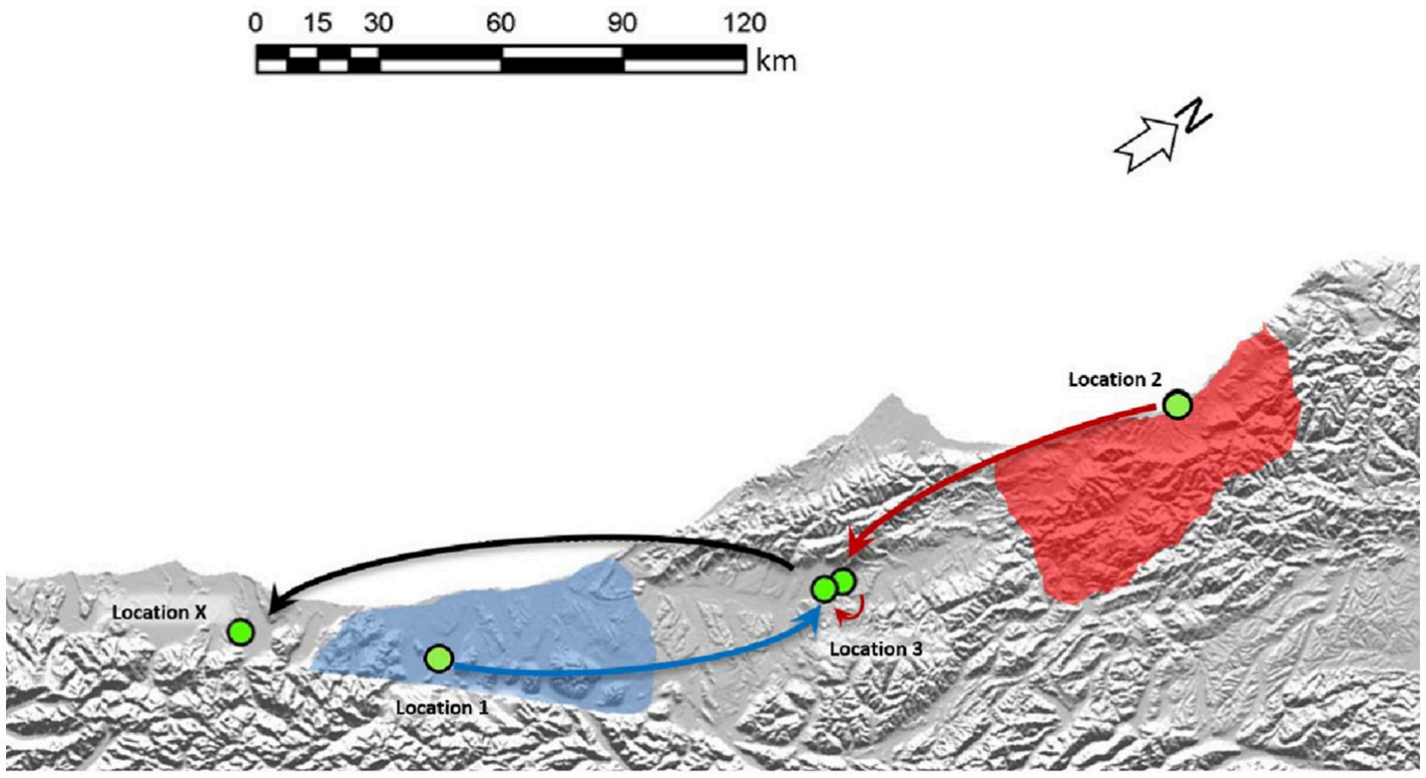
Two separate origins of TB in a South Westland Herd. Cattle movements that led to these two types of infection in the South Westland herd are indicated by arrows. Green circles indicate approximate locations where these animals resided. Shaded areas on the map indicate regions where VNTR53/REA11/REA12 (red) and VNTR59/REA1/REA6 (blue) are endemic in wildlife populations. TB case 1 moved from Location 1 to location 3 before moving to Location X (from where it was identified as TB positive at routine slaughter). TB case 2 was moved from its herd of origin in location 2 to a farm near Location 3 before moving to location 3 and then on to Location X from where it was identified as being infected. The region described here is indicated by the black box in the map in Figure 2A.

Results from the investigation into the findings of TB cases in a previously disease free dairy herd located in South Westland, West Coast, South Island are shown in Figures 4, 5 and Supplementary Files 3c,d, and 5). The herd had two separate findings of bovine tuberculosis approximately 5 months apart. There was a clear herd skin test between the two animals being identified at slaughter. Both TB cases were considered to be anergic animals (infected but not detectable through our standard testing procedures) as they were not identified as infected until they were inspected at slaughter, and both animals had been repeatedly TB skin tested before and after leaving their herd of origin.

These isolates were two distinct VNTR types; the first TB case (AgR738) was identified as type VNTR59, and the second case TB case (AgR744) was identified as type VNTR53 (see Figures 4A,B and Supplementary File 5). The Phylogram in Figure 4A illustrates the phylogenetic relationship of the isolates of these two types to others of these types in the database. This Phylogram was determined by ML analysis. The same relationship was evident by BioNJ analysis (see Supplementary Files 3c,d). The distinguishing SNPs in the SNP tables in Figure 4B provide a more detailed comparison of the differences between these TB isolates and their closest relatives. WGS clearly demonstrated the close relationship between the isolates and those from historic cases linked to the original locations of these animals. Animal movements were traced using information collected at the time of the epidemiological investigation. Animal identification and movement records were scrutinized as well as gathering information directly from farmers at that time. Although the two types that were detected in this herd could be distinguished from each other by VNTR assay, WGS analysis has allowed these to be compared to other isolates and confirm the most likely transmission pathway (see the square Phylograms in Supplementary File 5).

WGS clearly narrowed the list of likely suspects in each case. The isolate from the first TB case (AgR738) was identical or nearly identical by WGS to isolates from a recent outbreak up the coast in the Kowhitirangi and Arahura regions (see the SNP table Figure 4B). All of these outbreak isolates appeared to share a recent common ancestor with AgR288, a 2011 cattle beast isolate from a Hokitika farm, and this was thought to be a likely original source, but this isolate was ruled out as a source for the outbreak by WGS, since it was missing the 3 SNPs that are common to the outbreak and had additional SNPs not found in the outbreak isolates (Figure 4B). By WGS, the isolate from the second TB case (AgR744) was identical to AgR722, a livestock isolate from Westport, over 170 km from Location X and shared a recent common ancestor with several wildlife isolates (AgR296, AgR17133, AgR17134) from Karamea (location 2) which is over 250 km from Location X (see SNP tables in figure 4B and the map in Figure 5). Although all four locations on which the animals resided are within a formal Movement Control Area (MCA) where all stock over 12 months of age are to be tested annually AND all stock that are moved are to be TB tested within the 60 days preceding the movement, these results suggests that this infection has most likely resulted from the long distance movement of infected livestock.

### Waiuku outbreak investigation

**Figure 6:**
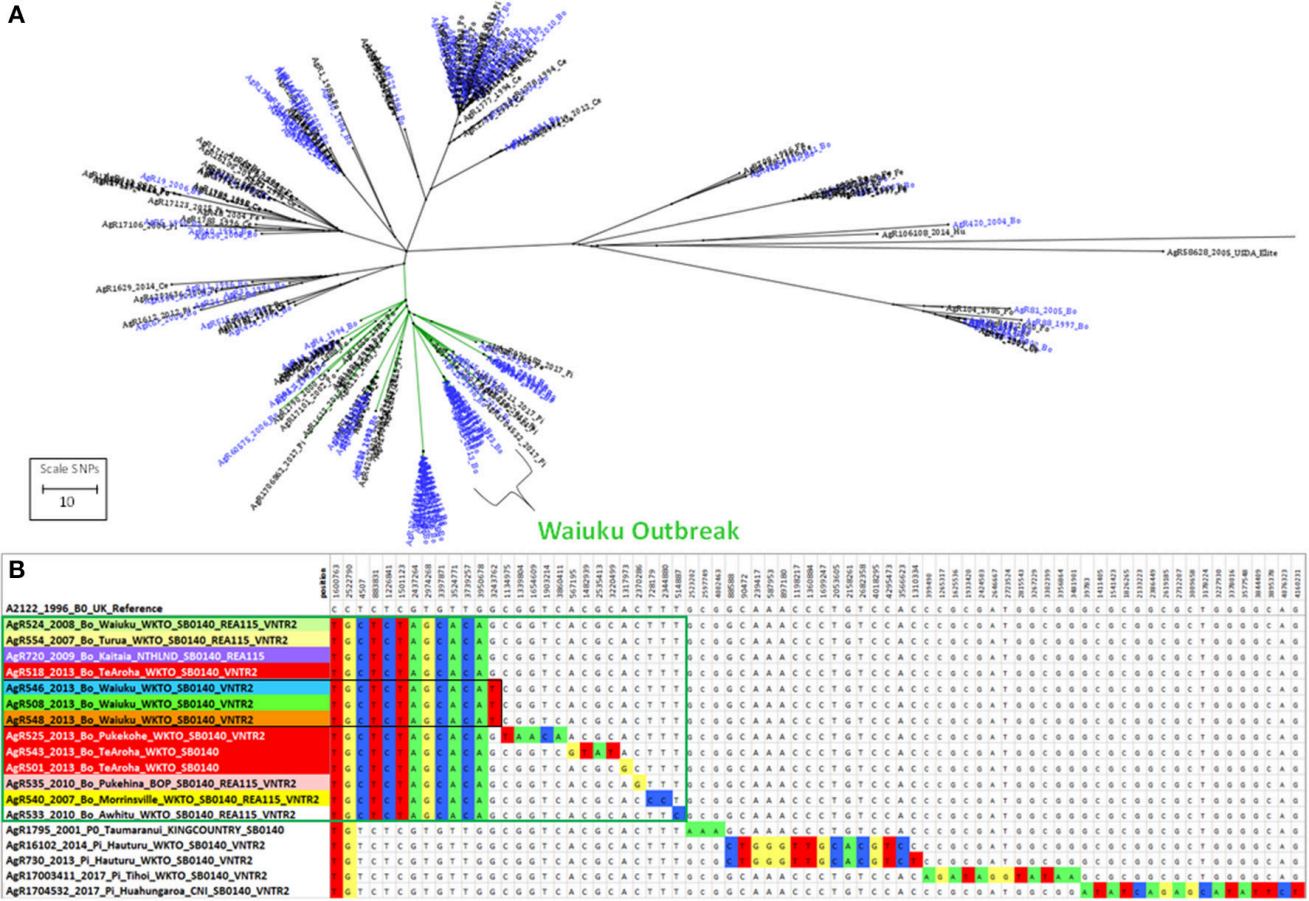
Genetic relationship of Waiuku outbreak Isolates. **(A)** Radial ML Phylogram illustrating the genetic relationship of *M. bovis* isolates from the Waiuku outbreak to other livestock and wildlife isolates in the Central North Island cluster. Livestock metadata are colored blue and wildlife metadata black. Waiuku isolates are indicated by the bracket. The green colored branch indicates the isolates that are compared in Supplementary File 3e. **(B)** The relationship of isolates from the Waiuku outbreak is illustrated in a SNP table with DNA bases in the table colored to indicate differences from the reference genome. Metadata for isolates from different herds that were characterized by WGS are shaded differently. Waiuku outbreak isolates characterized for this investigation are boxed in green. Isolates that are boxed in black were not known to be linked by movement but were from farms within 4 km of one another.

The investigation of the *M. bovis* outbreak that began in Waiuku, Central North Island clearly illustrated the close relationship of epidemiologically linked livestock isolates and demonstrated their more distant relationship to other types from the Central North Island (see the Phylogram in Figure 6A). The relationship of isolates in the green colored portion of the Phylogram in Figure 5A was compared by maximum likelihood and BioNJ methods (Supplementary File 3e) and found to be nearly identical by both methods.

**Figure 7:**
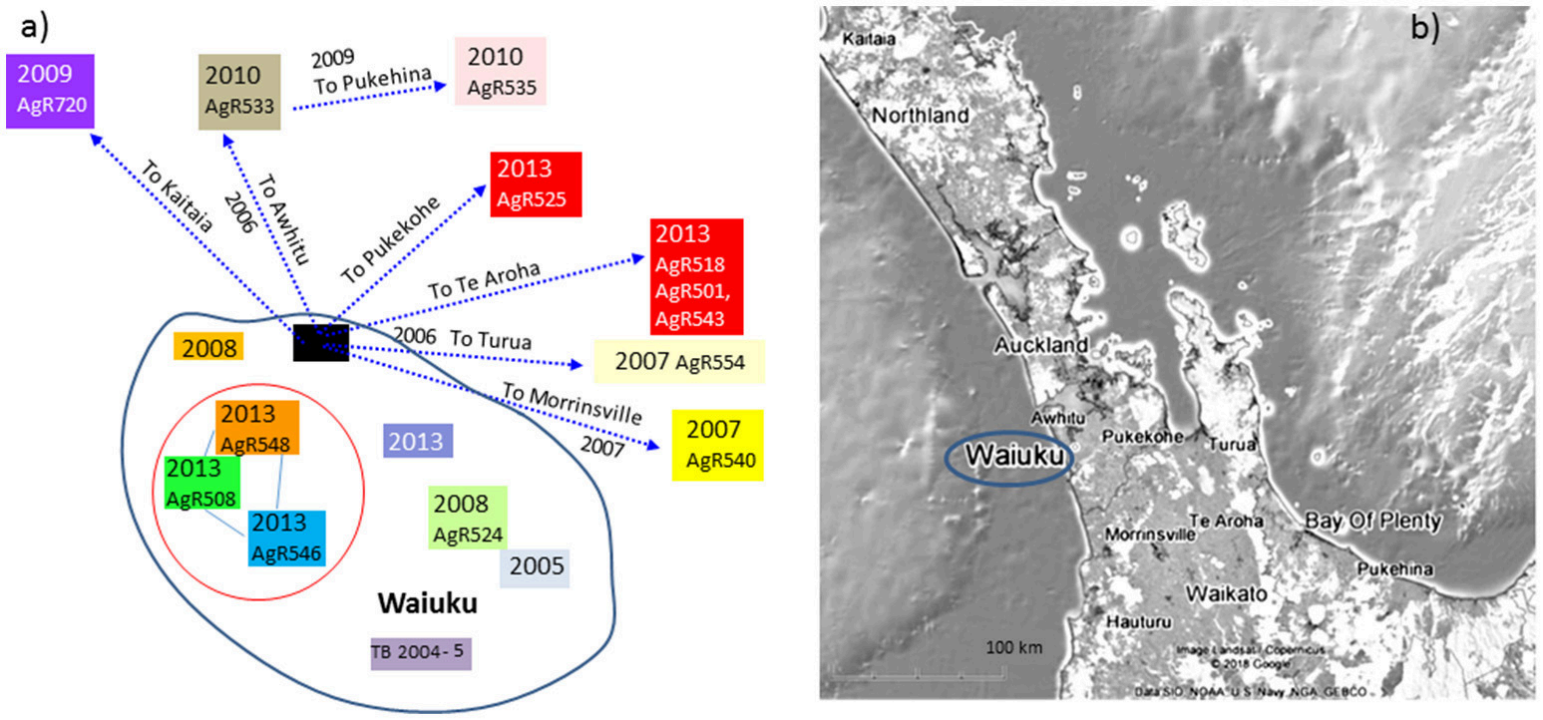
Waiuku outbreak Transmission path. **(a)** The direction of spread deciphered from epidemiological investigation, **(b)** a map illustrating the geographical sources of the characterized isolates. Isolates that have been characterized by WGS are colored to match the genomic data shown in the SNP table in Figure 6B. Colored boxes without AgR numbers represent isolates that were not characterized by WGS. Isolates that are circled in red were not known to be linked by movement but were from farms within 4 km of one another.

There were two cycles of infection associated with this outbreak, the first occurred between 2007 and 2010 and the second in 2013 (see Figure 7a). When compared by WGS, there were 10 SNPs detected that were common to both early and later outbreak isolates (see the SNP table in Figure 6B) suggesting that both outbreaks were from the same source of infection rather than from two different types introduced into the area. This infection was spread to herds in other regions of the Central North Island (Figure 7). Although no infection was detected from the likely source of this spread (black box in Figure 7a), isolates from infected animals that had been moved from this herd shared the 10 SNPs that were common to this outbreak (6b).

The relationship of Waiuku outbreak isolates to the closest known wildlife isolates in the database, recent pig isolates from Hauturu (AgR16102 and AgR730),Tihoi (AgR17003411) and Hauhungaroa (AgR1704532) as well as a possum isolate from 2001 (AgR1795) from Taumaranui, are also shown in the SNP table in Figure 6B. These results indicate that these wildlife isolates share a common ancestor with the outbreak isolates but they do not have the 10 outbreak specific SNPs and have acquired 13-15 SNPs that were not detected in the outbreak isolate genomes indicating that they are not closely related.

By combining epidemiological investigation with SNP lineage comparisons, far more insight is gained than was possible by VNTR or REA typing. A good example of this is provided by a SNP detected in isolates from livestock in Waiuku that were not known to be linked by movement but were farmed within 4 km of one another (AgR508, AgR546, and AgR548, boxed in black in the SNP table in Figure 6B and circled in the transmission path diagram in Figure 7) suggesting perhaps that despite extensive surveillance, there may have been either a wildlife vector for this Waiuku infection, or alternatively that there was undocumented herd movement occurring.

### Distance of epidemiologically linked isolates

**Figure 8:**
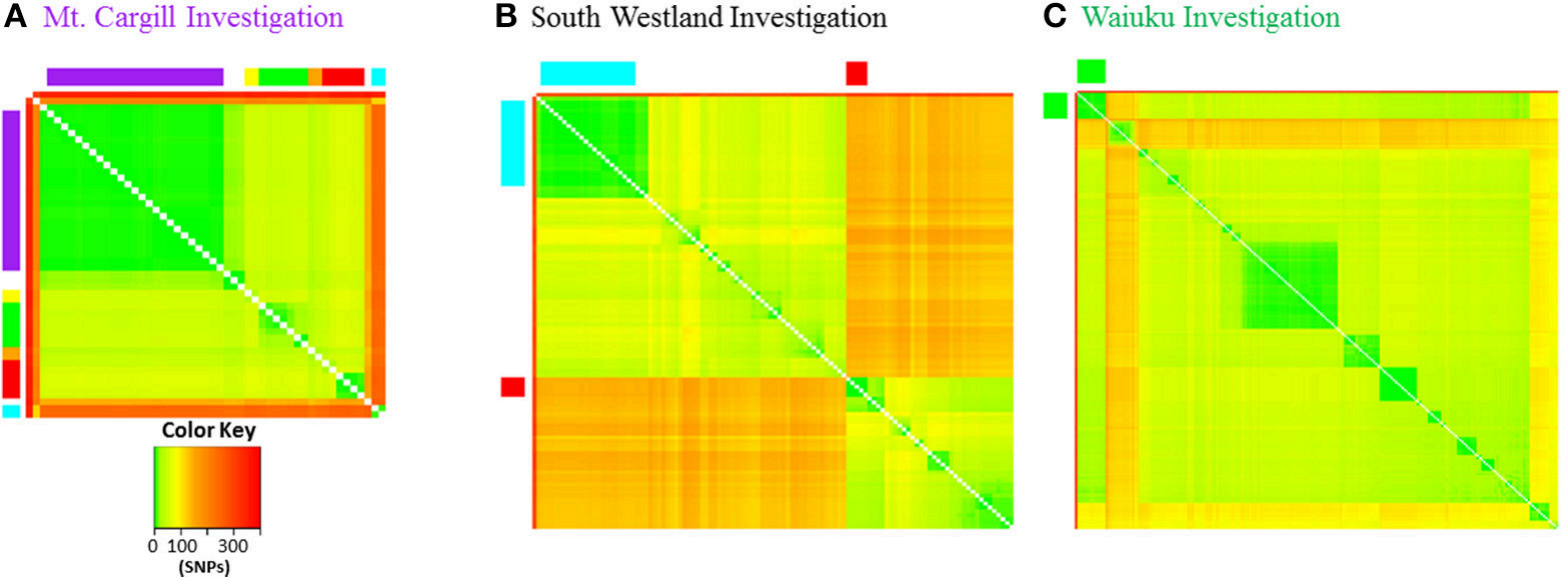
Pairwise genetic distance heat maps. Distance in SNPs between pairs of isolates is illustrated by the different colors as indicated in the color key. **(A)** Mt. Cargill outbreak isolates are in the same order as in the Phylogram in Figure 3 and colors along the outside of the plot correspond to those in the Phylogram and also to those in the more detailed distance plots in Supplementary File 6. **(B)** VNTR53 and VNTR59 isolates. Close relatives of South Westland breakdown type 1 are indicated by red squares and type 2 by the blue rectangles along the outside of the plot. Isolates are in the same order as in the more detailed distance plots in Supplementary File 6. **(C)** CNI branch isolates. Waiuku outbreak isolates are indicated by the green squares along the outside of the plot.

Genetic pairwise distances are the number of SNPs that differ when two isolates are compared. Table 1 compares pairwise distances for the epidemiologically linked isolates discussed above and the heat map in Figure 8 and in Supplementary File 6 show pairwise distances for all of the isolates illustrated for the three discussed investigations. Mt. Cargill isolates were collected over a period of 5 years and differed from one another by 0-5 SNPs. AgR 738, the case 2 isolate from the South Westland herd and its 22 close relatives from the Kowhitirangi outbreak were collected over a period of approximately 5 years, and differed from one another by 0–7 SNPs. The 12 Waiuku outbreak isolates were collected over a period of 6 years and differed from one another by 0–9 SNPs. The New Zealand *M. bovis* mutation rate determined by Crispell et al. (24) (0.53 with a range 0.22–0.94) was closest to that estimated for human tuberculosis by Walker et al. (44) (0.5 with a range of 0.3–0.7), and the pairwise distances of epidemiologically linked isolates shown in Table 1 are within the 12 SNP limit for epidemiological linkage determined by Walker and colleagues for *Mycobacterium tuberculosis*. These groups of isolates differ from unlinked isolates of the same types by 10's of SNPs and from isolates from other branches of the phylogenetic tree by hundreds of SNPs (see heat maps is Figure 8 and the more detailed versions in Supplementary File 6).

**Table 1:** Pairwise genomic distances of epidemiologically linked isolates.

**Outbreak**	**Number of isolates**	**Time span**	**Approx. # years**	**Pairwise distance (SNPs)**	**Hosts**	**VNTR**
Mt. Cargill	26	2012–2017	5–6	0–5	cattle, deer, possum	103
Kowhitirangi	23	2013–2018	5–6	0–7	cattle	59
Waiuku	12	2007–2013	6–7	0–9	cattle	2

## Discussion

Results of our current investigation demonstrated the same overall relationship of types described previously, since by WGS isolates cluster into the same groups that were determined by REA and VNTR analysis, but with the much finer resolution provided by WGS there is increased ability to rule out likely sources of infection. *In silico* spoligotyping confirmed that at least three of the four detected clades were likely to have been imported along with British sources of cattle in the middle to late 1800s. The regional clustering of types determined with REA and VNTR methods was corroborated since livestock and wildlife from the same region clustered. The Mt. Cargill and South Westland investigations illustrated how WGS leads to better definition of the source of new infections by ruling out potential sources, and all three investigations have led to the confirmation of epidemiological sourcing of infection. In addition, the Waiuku investigation indicated probable wildlife infection in an area considered to be free of infection.

The neighbor joining method is often considered useful for getting a quick approximate idea of genetic relationships because it is based principally on genetic distances, and does not incorporate the more accurate models of sequence evolution that are exploited in maximum likelihood analysis. The strikingly similar relationships determined for NZ *M. bovis* isolates by the maximum likelihood, neighbor joining distance methods and SNP tables, suggest that when analyzed by our WGS method, *M. bovis* in NZ cycles of animal infection appears to be evolving in a manner that is well described by incremental changes in genetic distance-clonal evolution. These results are in agreement with the evolutionary mechanism suggested in Smith et al. (45) where it was noted that these bacteria do not tend to carry or incorporate foreign DNA and that their genomes evolve primarily by deletions and the acquisition of SNPs.

The numerous SNPs that are shared by and distinguish members within and between groups give a much more robust indication of the relationship of isolates than our previous typing methods. In most cases the same REA and VNTR types tended to be grouped into one WGS cluster, but in several situations WGS revealed flaws in the apparent relationship of types determined by the other molecular methods. Several instances of homoplasy (the detection of the same REA or VNTR type in distantly related WGS clusters) were revealed during the course of our characterization of NZ types. For example, although most VNTR types tended to cluster into only one sub-group in the Otago branch, type VNTR27 isolates cluster in several different groups (see the phylogenetic tree in Figure 3b). This clustering of unrelated types has been described in other comparisons of WGS to VNTR typing (23, 46, 47). There were also several instances where types tended to switch back and forth within a subgroup, (see REA types 11 and 12 and REA types 1 and 6 in the tree in Supplementary Figure 5), as was observed by Trewby et al. (18).

Although WGS is far superior to our previous typing methods there are factors that limit the usefulness of WGS data for epidemiological investigations. *M. bovis* accumulates mutations in a clocklike manner, but the fixation of new changes into the population is slow and highly variable over short times. For an example see the SNP table in Figure 6B. Waiuku outbreak strains isolated within a year of one another varied by 0–5 SNPs. This variability has been noted in other *M. bovis* (16) and *M. tuberculosis* studies (48), and can make it difficult to determine whether the transmission is occurring within the herd or from local wildlife reinfection. This high variability over short times also makes it difficult to use Bayesian techniques such as BEAST for reconstructing recent local transmission pathways since there is not enough of a consistent temporal signal. Because the *M. bovis* lifestyle switches between an active systemic infection and a localized (difficult to detect) latent infection the time of infection is not necessarily close to the time of isolation. This makes it more difficult to determine when a new infection was introduced. When sampling disease with a wildlife reservoir the data may represent a low proportion of the total infection and it can therefore be difficult to draw valid conclusions about the direction of transmission. This influence of sampling bias was clearly illustrated in our previous work (24). In the current study, the finding of no close links between wildlife and Waiuku livestock may be because this type evolved in livestock populations and therefore there is no transmission linkage with wildlife but it could also be because the wildlife source was not sampled. This same factor weakens the conclusion drawn in the Otago study; although our analyses seem to indicate the infection in Mt Cargill came into the area in infected livestock, because of uneven sampling we cannot be 100% certain that the infection had not spread from an undetected local wildlife source.

The phylogeny in Figure 1 illustrates that NZ types share common ancestors with types isolated in other parts of the world [also see Supplementary File 4 and (43)]. We noted previously (24) that NZ strains tended to accumulate mutations at a faster rate than their UK relatives and surmised that the enhanced mutation rate may be the result of the larger amount of bacterial growth in possums, the major wildlife reservoir. Teasing apart these types of differences may be helpful for understanding transmission pathways in other bovine TB cycles.

## Conclusion

As the NZ epidemic diminishes, accuracy and high resolution becomes even more important for the identification of true sources. By ruling out possible sources of infection the enhanced resolution provided by WGS will likely reduce expenditure on the monitoring of herd infections and of wildlife monitoring and control. The routine use of WGS analyses for determining the source of *M. bovis* infections will be an important component of the strategy employed to eradicate bovine TB from NZ.

## Data availability

The datasets for this study have been deposited in the NCBI Sequence Read Archive (reference # SRP155672) and will be made publically available once this article has been accepted for publication.

## Author contributions

MP-C coordinated sequencing and data processing and drafted and revised the manuscript. DC, MP-C, GdL, and PL designed the analysis. RB developed data processing python scripts for mapping filtering and labeling genomic data. RB and MN developed figures. MN and JS shared results of epidemiological investigations, MN drafted a section of the manuscript, DC, GdL, PL, MN, JS, GK, GA, and KC were involved with selection of the isolates and interpretation of results. DC, GD, MP-C, and BP were involved with typing of isolates. JP, SH, and JW provided 200 sequences from Wellcome Sanger. RK provided 100 sequences from Wellcome Trust Glasgow. SR-A and TS shared reference genomes, helped with data filtering and data presentation. TS helped with installing and running vSNP software. DC, GdL, RB, BP GA RK SR-A JP, and JC critically reviewed and helped revise drafts of the manuscript. JP and MN helped with responses to reviewers.

### Conflict of interest statement

The authors declare that the research was conducted in the absence of any commercial or financial relationships that could be construed as a potential conflict of interest.

## Supplementary material

Supplementary File 1 SNPs excluded from the analysis. Numbers indicate regions or individual positions in UK reference version NC_002945.3 for which SNPs were excluded from all isolates.

Supplementary File 2 Isolate metadata, coverage statistics, *in silico* Spoligotype data and metadata explanations.

Supplementary File 3 Comparison of RaxML and BioNJ phylogenies. **(a)** NZ *M. bovis* tree. The four clades identified in Figure 1 with differently colored branches are boxed in the same color in this Figure. **(b)** Mt. Cargill outbreak investigation- all isolates shown in the Phylogram in Figure 3a are compared. Outbreak isolates are boxed in red. **(c-d)** South Westland investigation isolates are indicated by the red arrow, **(c)** case 1. **(d)** case 2. **(e)** Waiuku investigation. The scale indicates the level of relatedness between plots.

Supplementary File 4 Global distribution of NZ spoligotypes. Coloring in these plots reflects the relative abundance of this type in the indicated region.

Supplementary File 5 Square ML Phylogram illustrating the relationship of West Coast types considered for the South Westland investigations. Breakdown isolate metadata is colored red, livestock metadata is colored blue and wildlife metadata is colored black.

Supplementary File 6 Distance Matrices. Closely related isolates are colored green, more distant isolates yellow, then orange with the most distinct isolates colored red. Color bars indicate the corresponding cluster in Figures 3, 4 and 6.

## Abbreviations

*M. bovis*: *Mycobacterium bovis*
*M. tuberculosis*: *M. tuberculosis*
NZ: New Zealand
WGS: Whole Genome Sequencing
VNTR: Variable Number Tandem Repeat
REA: Restriction Enzyme Analysis
BEAST: Bayesian Evolutionary Analysis by Sampling Trees
AHVLA: Animal Health and Veterinary Laboratories Agency
USDA: United States Department of Agriculture
NZGL: New Zealand Genomics Limited
UK: United Kingdom
CTAB-N-cetyl-N: N,N-trimethyl ammonium bromide
BWA: Burrows Wheeler Aligner
OTGO: Otago
CNI: Central North Island
WC: West Coast
PPD: purified protein derivative
Bo: bovine
Po: possum
Pi: pig
Ce: cervine
Fe: ferret
ML: Maximum Likelihood.
