# Supplementary figures and images for: Whole Genome Sequencing for Determining the Source of Mycobacterium bovis Infections in Livestock Herds and Wildlife in New Zealand
Source: Front Vet Sci. 2018 Oct 30;5:272. doi: 10.3389/fvets.2018.00272 (PMC6218598; doi:10.3389/fvets.2018.00272)

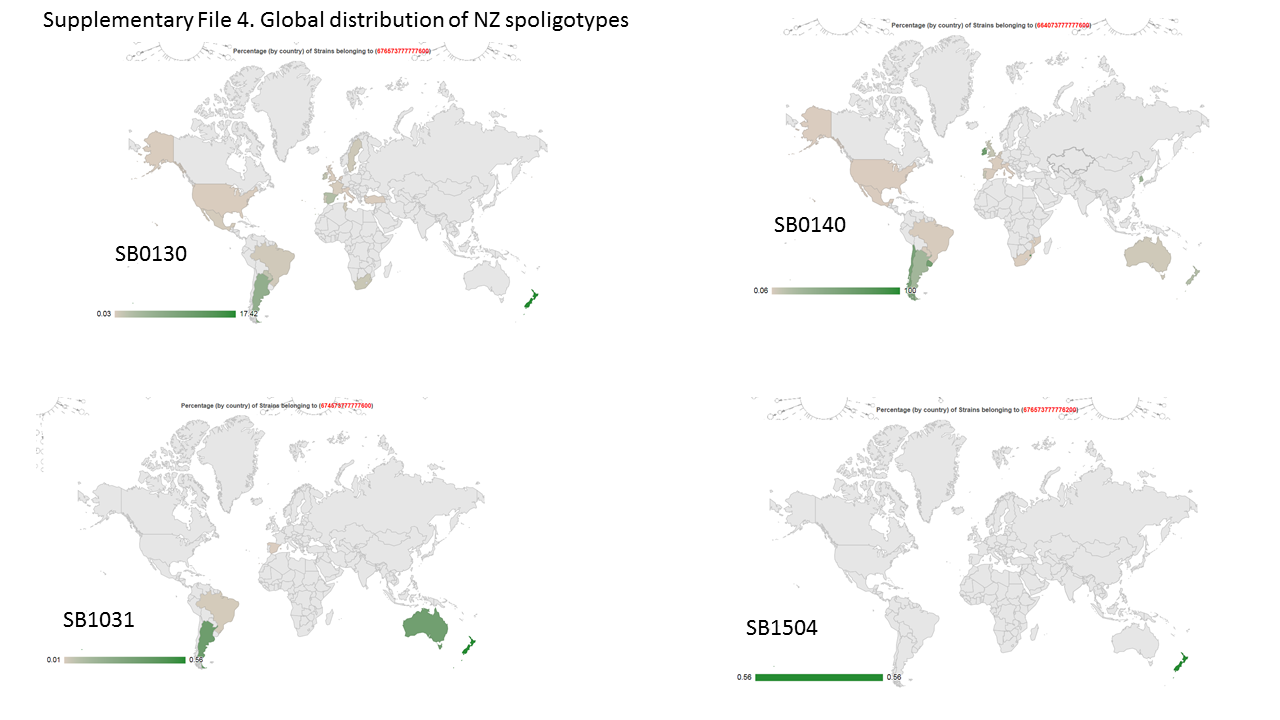

Supplement: Supplementary File 4 — Global distribution of NZ spoligotypes. Coloring in these plots reflects the relative abundance of this type in the indicated region. [file Image_1.TIF]

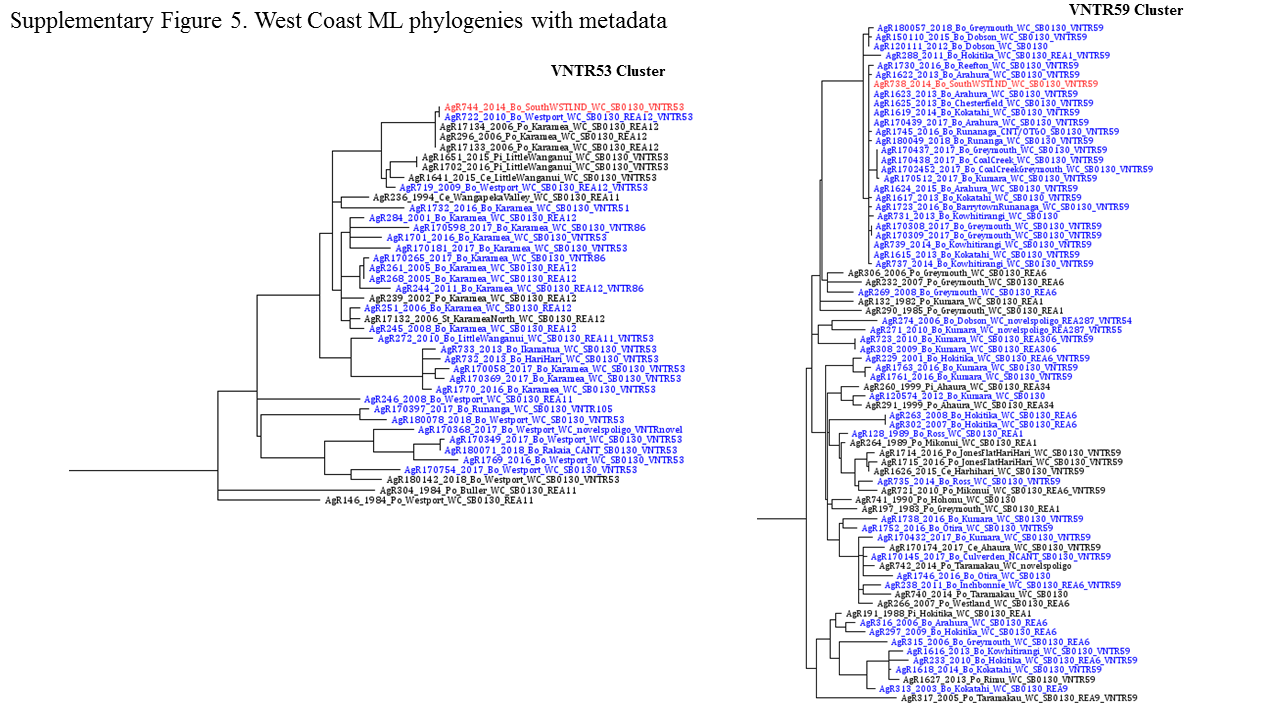

Supplement: Supplementary File 5 — Square ML Phylogram illustrating the relationship of West Coast types considered for the South Westland investigations. Breakdown isolate metadata is colored red, livestock metadata is colored blue and wildlife metadata is colored black. [file Image_2.TIF]

## Slide 1
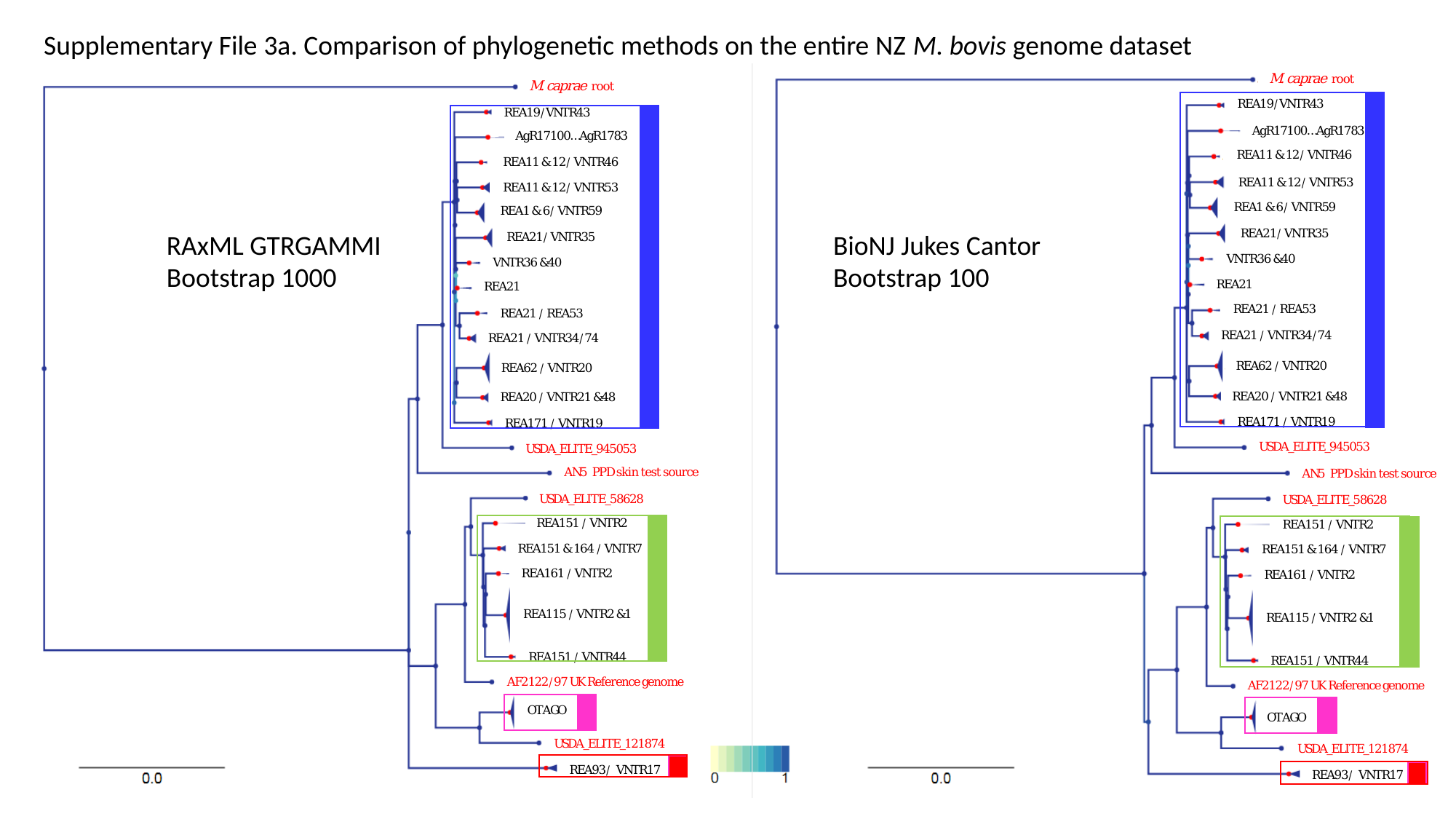

## Slide 2
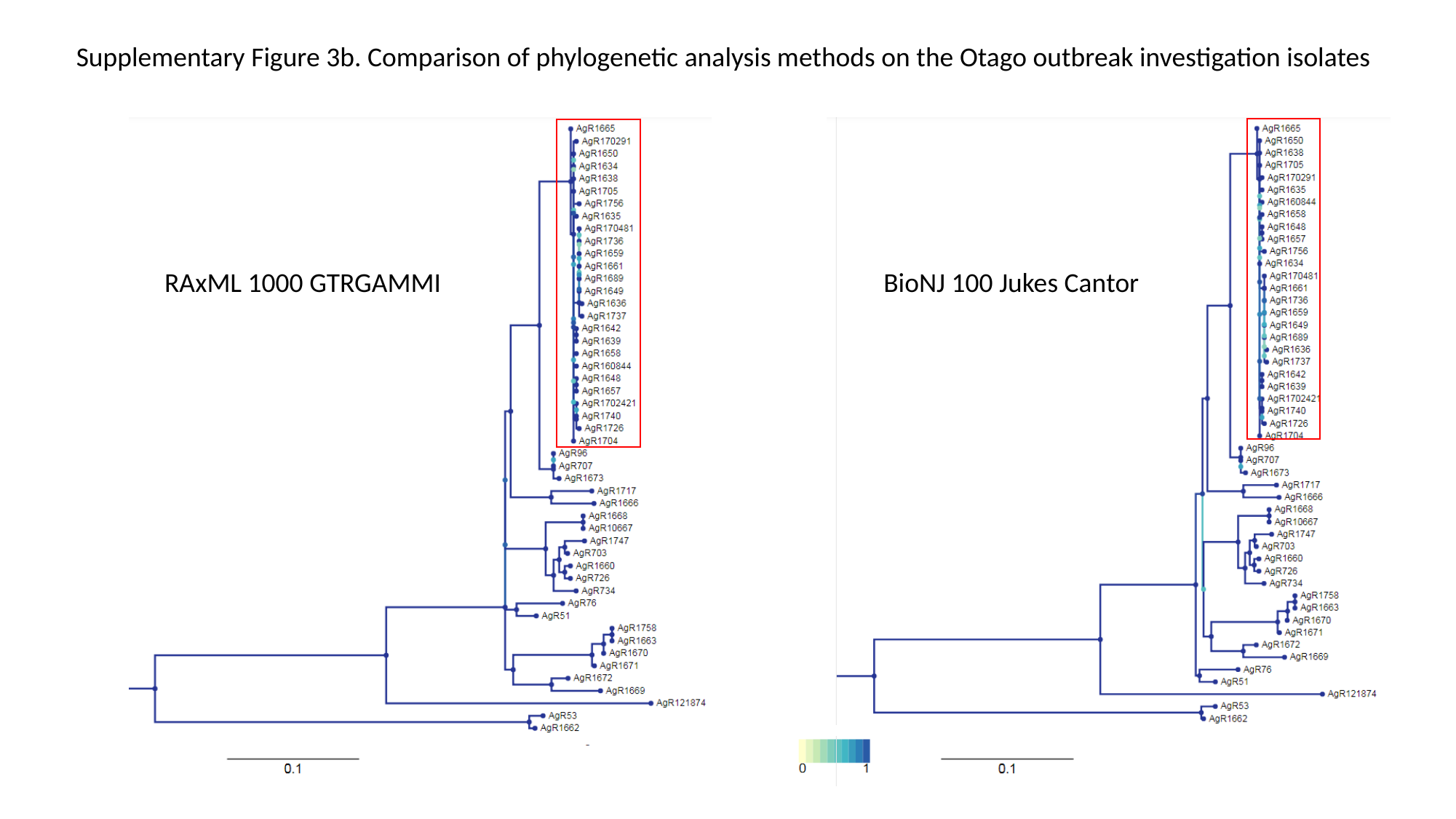

## Slide 3
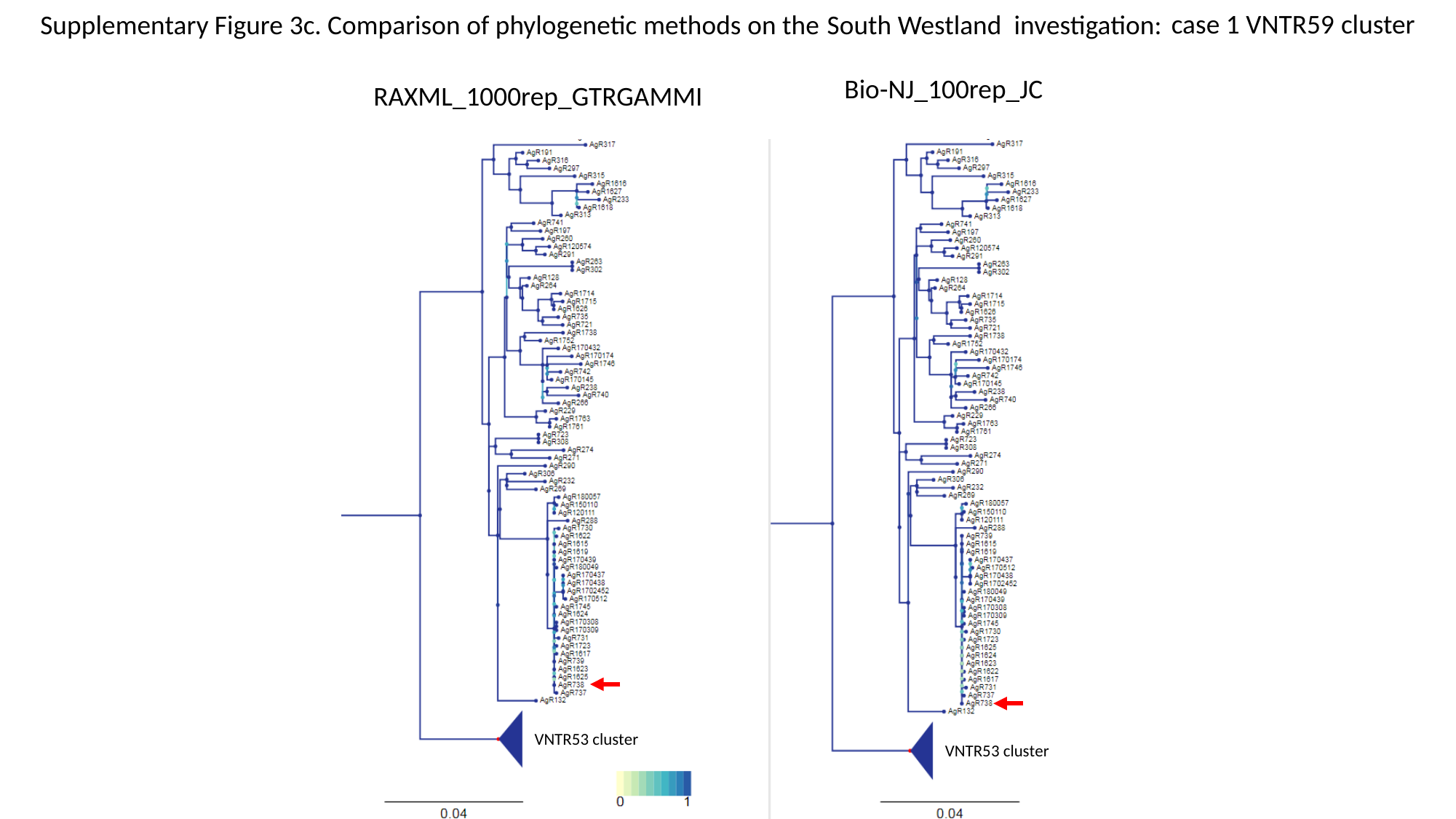

## Slide 4
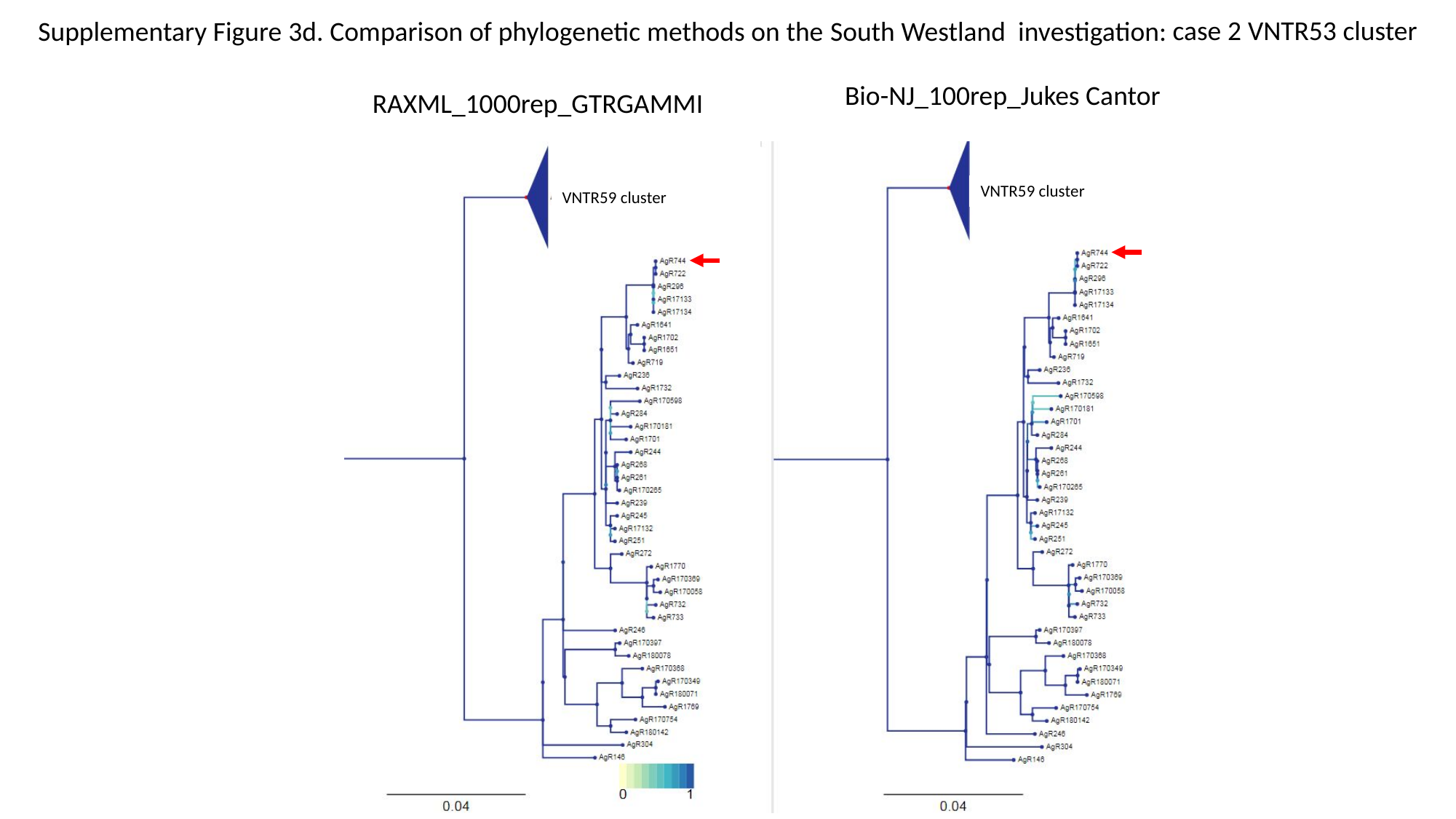

## Slide 5
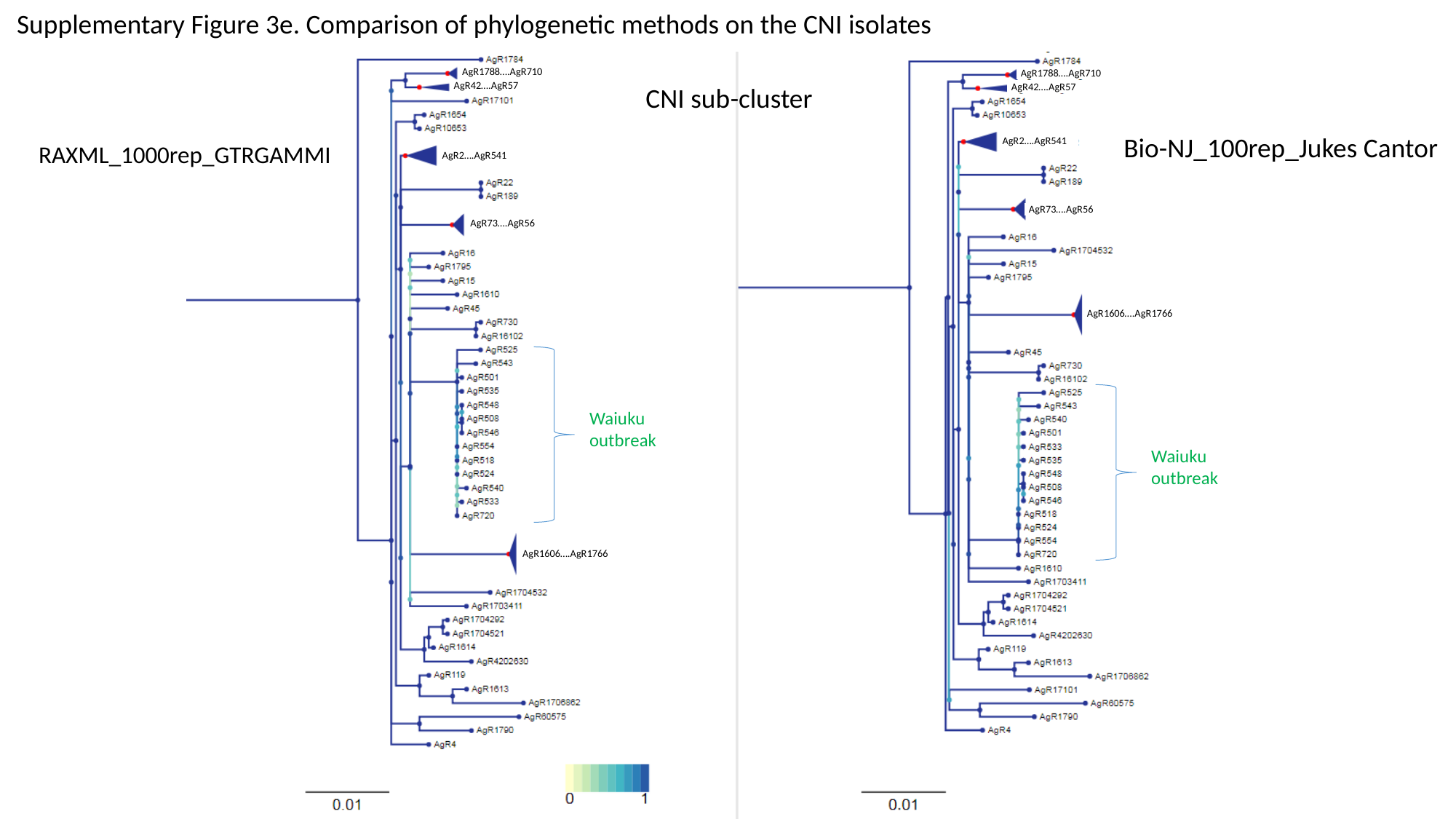

Supplement: Supplementary File 6 — Distance Matrices. Closely related isolates are colored green, more distant isolates yellow, then orange with the most distinct isolates colored red. Color bars indicate the corresponding cluster in Figures 3, 4 and 6. [file Presentation_1.PPTX]
